# Donor-derived Cell-free DNA Does Not Improve the Detection of Acute Kidney Allograft Rejection Beyond Urinary CXCL9 and CXCL10

**DOI:** 10.1097/FTD.0000000000001496

**Published:** 2026-07-08

**Authors:** Alvaro Assis de Souza, Annemiek Peeters, Douglas Bost, Carla Baan, Karin Boer, Dennis A. Hesselink

**Affiliations:** * Department of Internal Medicine, Division of Nephrology and Transplantation, Erasmus MC Transplant Institute, Erasmus University Medical Center Rotterdam, Rotterdam, the Netherlands; and; † JETA Molecular BV, the Netherlands.

## Abstract

Supplemental Digital Content is Available in the Text.

***To the Editor:***

Despite a strong interest of the transplantation community in the urinary chemokines CXCL9 and CXCL10 as noninvasive biomarkers for kidney allograft surveillance, their clinical utility for detecting allograft rejection and reducing adverse clinical outcomes in the first year post-transplant was found to be limited.^1,2^ One reason is their limited specificity for distinguishing alloimmune activity, as they can be elevated by other inflammatory conditions, including bacterial urinary tract infection and polyomavirus infection.

Donor-derived cell-free DNA (dd-cfDNA), a marker of allograft tissue injury, has also gained attention for use in the early detection of rejection and monitoring responses to antirejection therapy, with several analytical platforms developed for its quantification. Nevertheless, dd-cfDNA is also restricted about rejection specificity, as its concentrations may increase in response to other causes of tissue damage. Given the limited standalone performance of urinary CXCL9/CXCL10 and dd-cfDNA in detecting kidney allograft rejection, we asked the question whether their combined evaluation can improve such detection.

To answer said question, we concomitantly measured plasma dd-cfDNA and urinary CXCL9/CXCL10 levels at the time of for-cause kidney transplant biopsy after the first week post-transplant. Analyses were restricted to available paired samples (complete case analysis) from LIBIDAR, a prospective, observational, unselected cohort study conducted at the Erasmus MC University Medical Center Rotterdam.^3^ The study was approved by the local institutional review board (Medical Ethical Review Board Number 2018-035). Written informed consent was obtained from all patients.

Urinary CXCL9 and CXCL10 concentrations (pg/mL) were measured in duplicate, blinded, using commercial ELISA kits (CXCL9: Duoset DY392; CXCL10: Quantikine SIP100; R&D Systems; Minneapolis, MN). Plasma dd-cfDNA was quantified using HoloGRAFT (a droplet digital PCR-based assay; Omixon Biocomputing) and reported as fraction (%) and absolute (copies/mL) values. The dd-cfDNA fraction was used for the primary analysis and absolute values for the sensitivity analysis. Additional information on data processing and immunosuppressive protocols are summarized in **Supplemental Digital Content 1** (see **Supplementary Tables S1 and S2**, http://links.lww.com/TDM/A958).

To evaluate whether dd-cfDNA adds predictive value beyond urinary chemokines, we first fitted logistic regression models using log-transformed CXCL9 and CXCL10 values. We then added either dd-cfDNA fraction or absolute values to each model and assessed improvement in the model fit using likelihood ratio tests. Model discrimination was evaluated using the area under the receiver operating characteristic curve (AUC) with bootstrapping-based optimism correction. Outcomes were defined as (1) biopsy-proven rejection versus no rejection, excluding presumed rejection cases; and (2) treated versus untreated (treated = biopsy-proven acute rejection + presumed rejection). Presumed rejection was defined as clinically suspected rejection treated as rejection (usually with high-dose methylprednisolone) in the absence of definitive histological confirmation. It included cases in which biopsy was not feasible or showed negative or equivocal findings despite clinical suspicion. We accounted for multiple comparisons separately for the primary and sensitivity analyses. Statistical significance of model fit was defined as a Bonferroni-adjusted *P* < 0.05.

In total, 55 kidney transplant patients on a standard immunosuppressive regimen (tacrolimus, mycophenolic acid, and prednisolone) were included (see **Table S3**, **Supplemental Digital Content 1**, http://links.lww.com/TDM/A958 for baseline characteristics). The median time from transplantation to biopsy was 11 days (IQR: 8.0–54.5; see **Figure S1**; **Table S4**, **Supplemental Digital Content 1**, http://links.lww.com/TDM/A958). Of the 55 biopsies (1 per patient), 15 (27.3%) were histologically diagnosed as rejections, with 3 (5.5%) being antibody-mediated rejection (ABMR) and 12 (21.8%) T-cell–mediated rejection. Twenty-eight patients (50.9%) had no allograft rejections. Twelve patients (21.8%) classified under presumed rejection were considered a heterogeneous group, likely comprising both true but histologically unconfirmable rejection episodes and cases with an alternative and unproven cause of graft dysfunction. The distribution of prebiopsy tacrolimus predose concentrations showed no clear separation of diagnostic groups (see **Figure S2**, **Supplemental Digital Content 1**, http://links.lww.com/TDM/A958). Kidney function measures were restricted to patients not on dialysis at the time of for-cause biopsy.

Urinary CXCL9 and CXCL10 discriminated biopsy-proven rejection from no rejection, with AUCs of 0.79 (95% CI: 0.65–0.92) and 0.76 (95% CI: 0.53–0.89), respectively. The AUC for the dd-cfDNA fraction was 0.73 (95% CI: 0.54–0.88), whereas that for the absolute dd-cfDNA concentration was 0.71 (95% CI: 0.50–0.87). Combining the dd-cfDNA fraction with urinary CXCL9 or CXCL10 measures did not significantly improve the detection of kidney allograft rejection beyond either CXCL9 (delta AUC: 0.05 [95% CI: −0.08 to 0.17]) or CXCL10 (delta AUC: 0.01 [95% CI: −0.08 to 0.22]). Similarly, adding the dd-cfDNA fraction did not significantly improve the discrimination between treated and untreated patients beyond CXCL9 (delta AUC: 0.04 [95% CI: −0.04 to 0.11]) or CXCL10 (delta AUC: 0.02 [95% CI: −0.08 to 0.15]). Similar results were obtained for the sensitivity analysis with absolute dd-cfDNA values. The discrimination performance of all models and model fit likelihood ratio tests are summarized in Table 1. The diagnostic performance of dd-cfDNA at common thresholds is summarized in **Supplemental Digital Content 1** (see **Table S5**, http://links.lww.com/TDM/A958). The biomarker distributions are shown in **Supplemental Digital Content 1** (see **Figures S3 and S4**, http://links.lww.com/TDM/A958).

**TABLE 1.** Model Discrimination for Rejection and Need for Antirejection Treatment

	Rejection (n = 15) × No Rejection (n = 28)			Treated (Rejection + presumed Rejection; n = 27) × Not Treated (n = 28)		
	AUC (95% CI)	Delta AUC (95% CI)	Bonferroni-adjusted LRT *P*	AUC (95% CI)	Delta AUC (95% CI)	Bonferroni-adjusted LRT *P*
log CXCL9	0.79 (0.65–0.92)	NA	NA	0.74 (0.63–0.85)	NA	NA
log CXCL10	0.76 (0.53–0.89)	NA	NA	0.69 (0.52–0.82)	NA	NA
dd-cfDNA (%)	0.73 (0.54–0.88)	NA	NA	0.67 (0.51–0.79)	NA	NA
log dd-cfDNA (copies/mL)	0.71 (0.50–0.87)	NA	NA	0.66 (0.44–0.77)	NA	NA
log CXCL9 + dd-cfDNA (%)	0.84 (0.69–0.94)	0.05 (−0.08 to 0.17)	0.65	0.78 (0.66–0.89)	0.04 (−0.04 to 0.11)	0.52
log CXCL10 + dd-cfDNA (%)	0.77 (0.63–0.93)	0.01 (−0.08 to 0.22)	0.13	0.71 (0.56–0.83)	0.02 (−0.08 to 0.15)	0.13
log CXCL9 + log dd-cfDNA (copies/mL)	0.80 (0.63–0.92)	0.01 (−0.06 to 0.07)	1.00	0.75 (0.64–0.88)	0.01 (−0.05 to 0.01)	1.00
log CXCL10 + log dd-cfDNA (copies/mL)	0.77 (0.57–0.91)	0.01 (−0.07 to 0.15)	0.45	0.69 (0.53–0.82)	0.00 (−0.07 to 0.14)	0.37

Delta AUC represents the change in AUC after adding dd-cfDNA to the corresponding chemokine-only model. Improvement in the model fit by dd-cfDNA was evaluated using likelihood ratio tests for nested models. *P* are Bonferroni-adjusted.

LRT, log-likelihood ratio.

Our findings extend the discussion on the diagnostic limitations of dd-cfDNA in the early post-transplant period and help refine its clinical application. In our cohort, biopsies were limited to the first 6 months (median: 11 days; see **Figure S2**, **Supplemental Digital Content 1**, http://links.lww.com/TDM/A958). In this early post-transplant period, background injury from ischemia–reperfusion and perioperative events may reduce the rejection specificity of dd-cfDNA and bias its comparisons with urinary chemokines.^4^ Nevertheless, our findings are consistent with another study in which a model encompassing dd-cfDNA fraction and urinary chemokines in the context of for-cause biopsies also did not show improved performance (median: 17 months).^5^ Of note, we additionally assessed absolute dd-cfDNA values and observed similar results. We acknowledge that our limited sample size, especially the very low number of ABMR cases, may have reduced the power to detect the benefit of dd-cfDNA, a particularly informative marker for ABMR. In addition, T-cell–depleting induction therapy (n = 8; 14.6%) may have reduced the dd-cfDNA sensitivity in our cohort.

In conclusion, we found no evidence that dd-cfDNA improves the detection of kidney allograft rejection beyond urinary CXCL9 and CXCL10. Larger studies that go beyond AUC and incorporate model calibration and decision-analytic metrics are needed to determine whether combining biomarkers provides clinically meaningful improvements over reference chemokine models and whether distinct chemokine/dd-cfDNA interaction patterns reflect clinically relevant states of immune activation and tissue injury.

## Supplementary Material
