# Supplementary material for: Donor-derived Cell-free DNA Does Not Improve the Detection of Acute Kidney Allograft Rejection Beyond Urinary CXCL9 and CXCL10
Source: Ther Drug Monit. 2026 Jul 8;48(5):709–11. doi: 10.1097/FTD.0000000000001496 (PMC13557482; doi:10.1097/FTD.0000000000001496)
Supplement: Supplementary file 1 [file tdm-48-709-s001.docx]

**SUPPLEMENTARY DIGITAL CONTENT**

**Donor-derived Cell-free DNA Does Not Improve the Detection of Acute Kidney Allograft Rejection Beyond Urinary CXCL9 and CXCL10**

Alvaro Assis de Souza*, Annemiek Peeters*, Douglas Bost^†^, Carla Baan*, Karin Boer*, Dennis A. Hesselink*

*Department of Internal Medicine, Division of Nephrology and Transplantation, Erasmus MC Transplant Institute, Erasmus University Medical Center Rotterdam, Rotterdam, the Netherlands

^†^JETA Molecular BV, the Netherlands

Table of Contents

[SUPPLEMENTARY METHODS	3](#_Toc229828214)

[Calculation of chemokine concentrations	3](#_Toc229828215)

[Immunosuppression protocol	3](#_Toc229828216)

[Table S1. Distribution of CXCL9 Measurements Relative to the Assay Quantification Range, Stratified by Biopsy Clinical Diagnosis	5](#_Toc229828217)

[Table S2. Distribution of CXCL10 Measurements Relative to the Assay Quantification Range, Stratified by Biopsy Clinical Diagnosis	6](#_Toc229828218)

[Figure S1. Histogram of the timing of biopsies	7](#_Toc229828219)

[Figure S2. Scatter plot of days of tacrolimus measurement before biopsy versus tacrolimus pre-dose concentration.	8](#_Toc229828220)

[Figure S3. Scatter plot of log CXCL9 versus cell-free DNA fraction.	9](#_Toc229828221)

[Figure S4. Scatter plot of log CXCL10 versus cell-free DNA fraction.	10](#_Toc229828222)

[SUPPLEMENTARY RESULTS	11](#_Toc229828223)

[Table S3. Baseline Patient Characteristics	11](#_Toc229828224)

[Table S4. Findings at the Time of For-Cause Biopsies	13](#_Toc229828225)

[Table S5. Diagnostic Performance of dd-cfDNA at Common Clinical Thresholds	14](#_Toc229828226)

# SUPPLEMENTARY METHODS

## Calculation of chemokine concentrations

Standard curves were generated by plotting absorbance values against chemokine concentrations from kit control samples and fitting the data with a four-parameter logistic model according to the manufacturer instructions. The CXCL9 and CXCL10 concentrations were obtained by inverse prediction (interpolation) from the fitted curves within the assay ranges (CXCL9: 62.5–4000 pg/mL; CXCL10: 7.81–500 pg/mL). Because concentrations cannot be derived for absorbance values outside of the standard-curve range, out-of-range results were treated as censored: values below the lower limit were assigned half of the lower limit, and values above the upper limit were assigned the upper limit.

## Immunosuppression protocol

Maintenance immunosuppression comprised twice-daily immediate-release tacrolimus, mycophenolic acid, and prednisolone. Tacrolimus was initiated at 0.2 mg·kg^–1^·day^–1^, targeting a pre-dose (or “trough”; C_0_) concentration of 8–12 ng/mL during the first 4 weeks after transplantation, 5–10 ng/mL from weeks 5 to 12, and 5–8 ng/mL from months 3 to 6. Mycophenolic acid was administered at 1000 mg twice daily, with target pre-dose concentrations of 1.5–3 mg/L throughout the first 6 months. Prednisolone was given orally at 20 mg once daily from postoperative days 4 to 14; the dose was then reduced to 15 mg once daily during weeks 3–4, further reduced to 10 mg during weeks 5–6 and to 7.5 mg during weeks 7–12, and then maintained at 5 mg daily during months 3–4, after which it was tapered and discontinued by month 5. Additionally, valganciclovir prophylaxis was prescribed according to donor/recipient cytomegalovirus serostatus: no prophylaxis for D-/R-, 6 months for D+/R-, and 3 months for D+/R+ and D-/R+, extended to 6 months in case of severe immunosuppression. Dosing was adjusted according to kidney function: 900 mg once daily for eGFR >60 mL/min, 450 mg once daily for eGFR 40–59 mL/min, 450 mg every other day for eGFR 25–39 mL/min, and 450 mg twice weekly for eGFR 10–24 mL/min. Patients with eGFR <10 mL/min were monitored.

## Table S1. Distribution of CXCL9 Measurements Relative to the Assay Quantification Range, Stratified by Biopsy Clinical Diagnosis

| **Diagnosis** | **Above Range** | **Below Range** | **In Range** |
| --- | --- | --- | --- |
| ABMR | 1 | 2 | 0 |
| TCMR | 1 | 3 | 8 |
| Presumed rejection | 0 | 6 | 6 |
| ATN/Other (no rejection) | 0 | 22 | 6 |
| Total | 2 | 33 | 20 |

ABMR, antibody-mediated rejection; TCMR, T-cell-mediated rejection; ATN, acute tubular necrosis.

## Table S2. Distribution of CXCL10 Measurements Relative to the Assay Quantification Range, Stratified by Biopsy Clinical Diagnosis

| **Diagnosis** | **Above Range** | **Below Range** | **In Range** |
| --- | --- | --- | --- |
| ABMR | 1 | 1 | 1 |
| TCMR | 1 | 0 | 11 |
| Presumed rejection | 0 | 2 | 10 |
| ATN/Other (no rejection) | 0 | 5 | 23 |
| Total | 2 | 8 | 45 |

ABMR, antibody-mediated rejection; TCMR, T-cell-mediated rejection; ATN, acute tubular necrosis.

## Figure S1. Histogram of the timing of biopsies

## Figure S2. Scatter plot of days of tacrolimus measurement before biopsy versus tacrolimus pre-dose concentration.

## Figure S3. Scatter plot of log CXCL9 versus cell-free DNA fraction.

## Figure S4. Scatter plot of log CXCL10 versus cell-free DNA fraction.

# SUPPLEMENTARY RESULTS

## Table S3. Baseline Patient Characteristics

| **Variables** | **n** | **Values (n = 55)** |
| --- | --- | --- |
| **Recipient characteristics** |  |  |
| Age (years) | 55 | 63.0 (51.5–68.0) |
| Sex | 55 |  |
| Female |  | 25 (45.5%) |
| Male |  | 30 (54.5%) |
| Cause of end-stage kidney disease | 55 |  |
| Polycystic kidney disease |  | 6 (10.9%) |
| Diabetic nephropathy |  | 8 (14.5%) |
| Hypertensive nephropathy |  | 4 (7.3%) |
| Glomerulonephritis |  | 14 (25.5%) |
| Reflux nephropathy / Chronic pyelonephritis |  | 6 (4.8%) |
| Other |  | 20 (36.4%) |
| Unknown |  | 3 (5.5%) |
| **Donor characteristics** |  |  |
| Age (years) | 55 | 61.0 (53.0–66.0) |
| Sex | 55 |  |
| Female |  | 29 (52.7%) |
| Male |  | 26 (47.3%) |
| Type | 55 |  |
| Living |  | 24 (43.6%) |
| Deceased after brain death |  | 11 (20.0%) |
| Deceased after circulatory death |  | 20 (36.4%) |
| **Transplant baseline characteristics** |  |  |
| Total number of kidney transplantations | 55 |  |
| 1 |  | 42 (76.4%) |
| 2 |  | 10 (18.2%) |
| 3 |  | 2 (3.6%) |
| 4 |  | 1 (1.8%) |
| Delayed graft function | 55 | 22 (40.0%) |
| HLA A/B/DR mismatches | 55 |  |
| 0 |  | 3 (5.5%) |
| 1 |  | 2 (3.6%) |
| 2 |  | 10 (18.2%) |
| 3 |  | 14 (25.5%) |
| 4 |  | 9 (16.4%) |
| 5 |  | 9 (16.4%) |
| 6 |  | 8 (14.5%) |
| ABO incompatible transplantation | 55 | 4 (7.3%) |
| Historic panel reactive antibody | 48 | 4.0 (0.0–47.8) |
| Current panel reactive antibody | 50 | 0.0 (0.0–4.0) |
| Cold ischemia time (min) | 31 | 717.0 (550.5–910.0) |
| Organ preservation | 31 |  |
| Static cold storage |  | 6 (10.9%) |
| Hypothermic machine perfusion |  | 18 (32.7%) |
| Normothermic machine perfusion |  | 5 (9.1%) |
| Unknown |  | 2 (3.6%) |
| Induction therapy | 55 |  |
| No induction |  | 1 (1.8%) |
| Basiliximab |  | 46 (83.6%) |
| Anti-thymocyte globulin |  | 3 (5.5%) |
| Alemtuzumab |  | 5 (9.1%) |

## Table S4. Findings at the Time of For-Cause Biopsies

| **Variables** | **n** | **Values (n = 55)** |
| --- | --- | --- |
| Time of biopsy after transplantation, days | 55 | 11.0 (8.0–54.5) |
| Patient group and histologic diagnosis | 55 |  |
| Acute rejection (histologic rejection classification) |  |  |
| aABMR |  | 3 (5.5%) |
| aTCMR1B |  | 2 (3.6%) |
| aTCMR2A |  | 5 (9.1%) |
| aTCMR2B |  | 3 (5.5%) |
| aTCMR2B plus recurrent IgAN |  | 1 (1.8%) |
| aTCMR3 |  | 1 (1.8%) |
| Presumed rejection (histologic findings)^a^ |  |  |
| Acute tubular necrosis |  | 4 (7.3%) |
| Acute thrombotic microangiopathy |  | 2 (3.6%) |
| Microvascular inflammation not otherwise specified |  | 2 (3.6%) |
| Diabetic nephropathy |  | 1 (1.8%) |
| Recurrent IgA nephropathy and diabetic nephropathy |  | 1 (1.8%) |
| Collapsing focal segmental glomerulosclerosis |  | 1 (1.8%) |
| Urinary tract infection |  | 1 (1.8%) |
| No rejection (histologic findings) |  |  |
| Acute tubular necrosis |  | 20 (36.4%) |
| Acute tubular necrosis and donor-derived arteriolar hyalinosis |  | 1 (1.8%) |
| Arteriolar hyalinosis |  | 3 (5.5%) |
| Transplant pyelonephritis |  | 1 (1.8%) |
| Acute pyelonephritis |  | 1 (1.8%) |
| Borderline TCMR |  | 1 (1.8%) |
| Normal light microscopy findings |  | 1 (1.8%) |
| Serum creatinine concentration at time of biopsy, µmol/L^b^ | 29 | 185.0 (160.0–237.0) |
| Estimated glomerular filtration rate at time of biopsy, mL/min^b^ | 29 | 29.0 (20.0–36.0) |

^a^ Patients who were considered in need of anti-rejection therapy despite inconclusive or negative histologic findings for rejection during histopathological assessment (shown on increased indented level). ^b^ Only for patients not on dialysis at the time a biopsy was performed. ABMR, antibody-mediated rejection; TCMR, T-cell-mediated rejection.

## Table S5. Diagnostic Performance of dd-cfDNA at Common Clinical Thresholds

|  | Rejection (n = 15) x No Rejection (n = 28) | | | | Treated (rejection + presumed rejection; n = 27) x Not treated (n = 28) | | | |
| --- | --- | --- | --- | --- | --- | --- | --- | --- |
| Threshold (%) | Sensitivity | Specificity | PPV | NPV | Sensitivity | Specificity | PPV | NPV |
| 0.5 | 0.67 | 0.68 | 0.53 | 0.79 | 0.59 | 0.68 | 0.64 | 0.63 |
| 1 | 0.33 | 0.86 | 0.56 | 0.71 | 0.30 | 0.86 | 0.67 | 0.56 |

PPV, positive predictive value; NPV, negative predictive value.
